# Data on the drug release profiles and powder characteristics of the ethyl cellulose based microparticles prepared by the ultra-fine particle processing system

**DOI:** 10.1016/j.dib.2020.105269

**Published:** 2020-02-08

**Authors:** Tingting Peng, Yin Shi, Chune Zhu, Disang Feng, Xiangyu Ma, Peipei Yang, Xuequn Bai, Xin Pan, Chuan-yu Wu, Wen Tan, Chuanbin Wu

**Affiliations:** a School of Pharmaceutical Sciences, Sun Yat-sen University, Guangzhou, 510006, People's Republic of China; b Institute for Biomedical and Pharmaceutical Sciences, Guangdong University of Technology, Guangzhou, 510006, People's Republic of China; c College of Pharmacy, University of Texas at Austin, Austin, 78712, USA; d Department of Chemical and Process Engineering, University of Surrey, Guildford, Surrey, GU2 7XH, United Kingdom

**Keywords:** Microparticles, Drug release profile, Powder characteristic

## Abstract

Ethyl cellulose (EC) based microparticles (MPs) could provide sustained release for Huperzine A. The drug release mechanism of MPs was exploited to achieve an ideal drug release profile. We previously found that the wettability of MPs greatly contributed to facilitating drug release, which was detailed in a research article entitled “Huperzine A loaded multiparticulate disintegrating tablet: Drug release mechanism of ethyl cellulose microparticles and pharmacokinetic study” (Peng et al., 2019) [1]. In this article, the influence of different polymers and drugs on the drug release behavior was investigated to broaden or compensate this finding. Besides, powder characterization of MPs was used to evaluate the further application of MPs for tablets.

**Table:** Specifications Table

Subject area	Pharmaceutics
More specific subject area	Controlled drug delivery system
Type of data	Tables, figures
How data was acquired	ZRS-8G dissolution tester (TDTF, Tianjin, China) and High performance liquid chromatography (LC-20, Shimadzu, Japan) FT4 Powder Rheometer (FreemanTechnology, Tewkesbury, UK)
Data format	Raw, descriptive and inferential
Experimental factors	Drug dissolution study: (ⅰ) type of dissolution media, (ⅱ) type of polymer, and (ⅲ) type of model drug Powder characterization: source of powder
Experimental features	Evaluate the formulation and media compositions on the drug release profiles of different MPs Compare the difference in the powder characteristics of developed MPs and commercial excipient
Data source location	School of Pharmaceutical Sciences, Sun Yat-sen University, Guangzhou, 510006, China
Data accessibility	Data is with this article.
Related research article	T. Peng, Y. Shi, C. Zhu, D. Feng, X. Ma, P. Yang, X. Bai, X. Pan, CY. Wu, W. Tan, C. Wu. Huperzine A loaded multiparticulate disintegrating tablet: Drug release mechanism of ethyl cellulose microparticles and pharmacokinetic study. Powder Technology. 2019, 355:649–656 [1].

**Table:** 

**Value of the Data** • The data on how the polymers and model drugs influenced the drug release behavior can be compared with the other results detailed in our published research [1], which better illuminates the drug release mechanism of microparticles (MPs) and provides scientific guidance on formulation development. • The data obtained from the tests by FT4 Powder Rheometer provides useful information on the powder characteristics of MPs, which are closely related to the compressibility of MPs. • This data provides guidance for the pharmaceutical industry to develop multiparticulate tablets with MP as a subunit.

## 1 Data

**Fig. 1:**
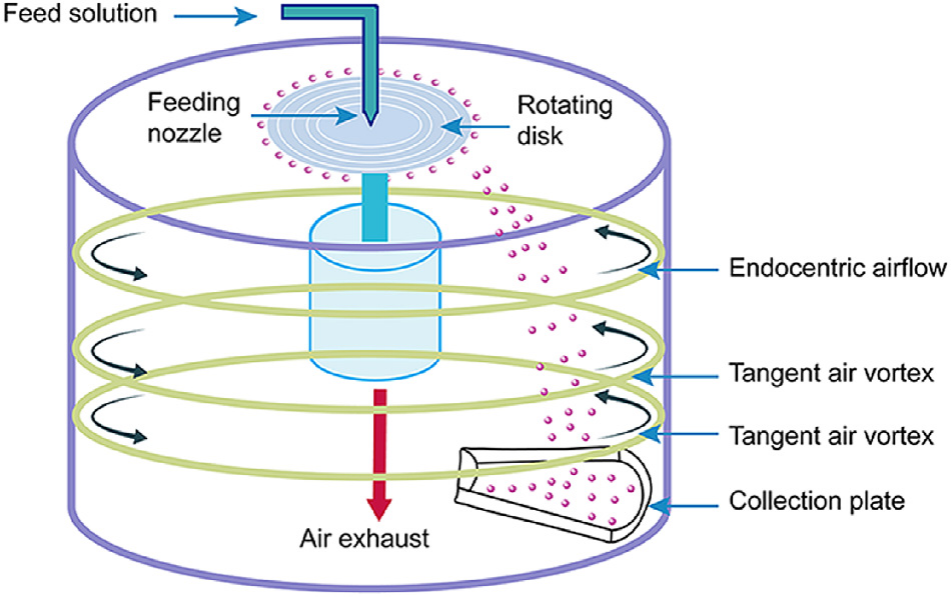
The schematic illustration of UPPS apparatus for MPs production, which was modified from a previous literature [4].

**Fig. 2:**
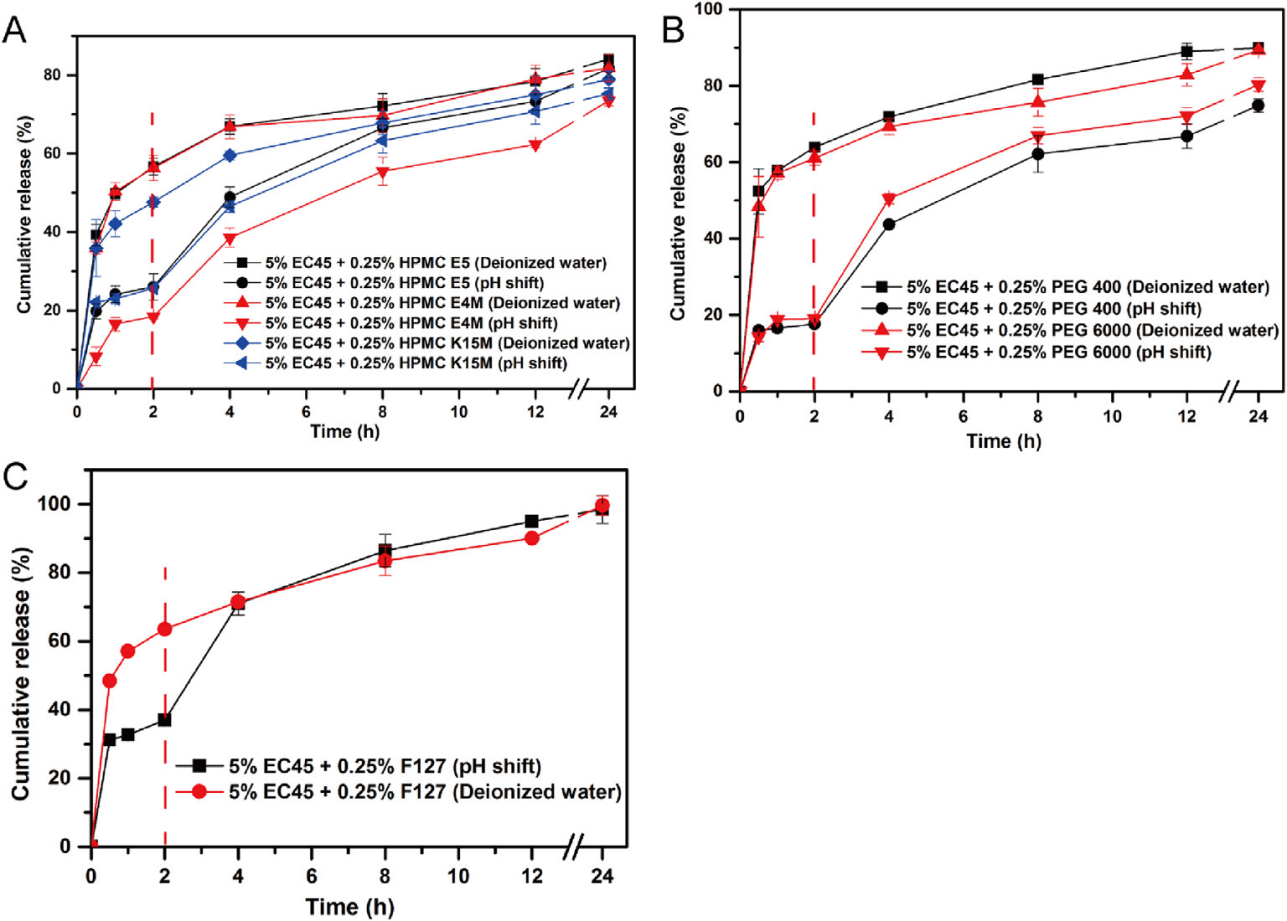
The influence of different additives on drug release profiles of EC MPs loading HupA (*n* = 3): (A) HPMC grade polymers, (B) PEG grade polymers, and (C) amphiphilic polymer F127.

**Fig. 3:**
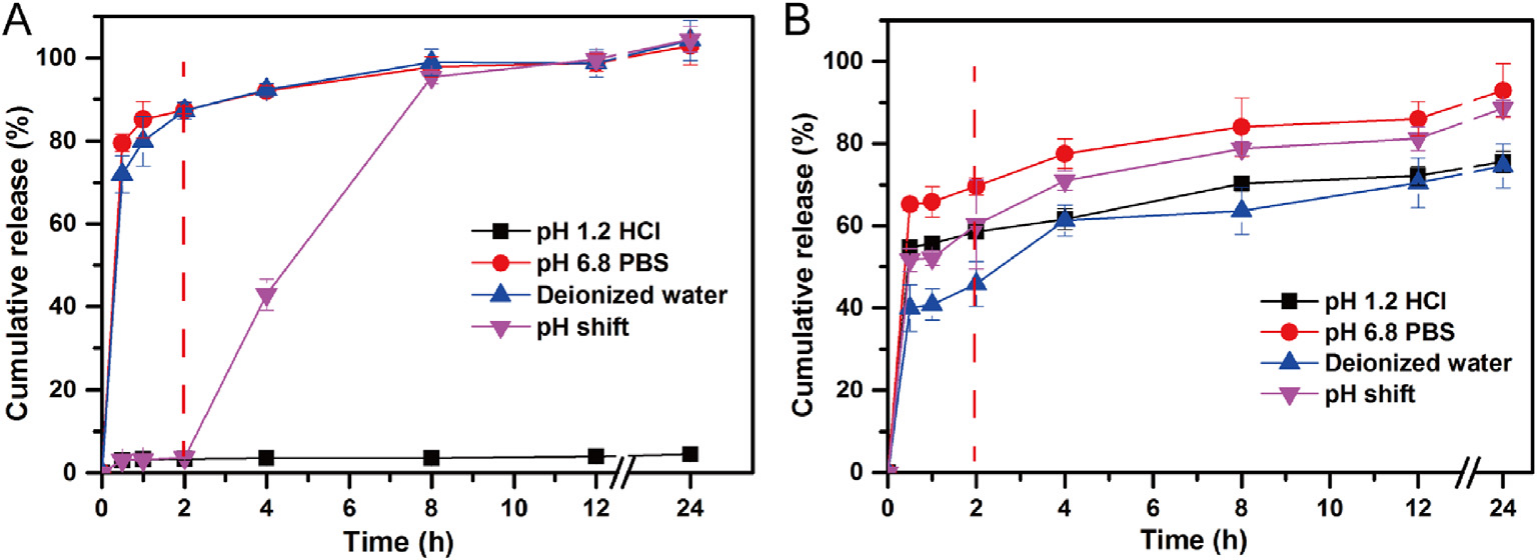
The influence of dissolution medium on (A) weak acid drug (ketoprofen) and (B) water-soluble drug (ciprofloxacin hydrochloride) loaded EC MPs (*n* = 3).

The data in subsection 2.1 displayed the schematic illustration (Fig. 1) of ultra-fine particle processing system (UPPS) for preparing MPs. In subsection 2.2, we displayed the data regarding to the comparative influence of water-soluble or amphiphilic polymers (Fig. 2) and model drugs (Fig. 3) on the release profiles of EC based MPs under the simulated gastrointestinal fluid and deionized water, respectively. In subsection 2.3, the data (Table 1) summarized the main parameters of MPs and Ludiflash® obtained from the flow energy test.

**Table 1:** The main parameters of MPs and Ludiflash® obtained from the flow energy test (*n* = 3).

Material	*BFE* (mJ)	*SI*	*FRI*	*SE* (mJ/g)	*CBD* (g/ml)
Ludiflash®	220.41 ± 1.53	1.03 ± 0.01	1.27 ± 0.03	5.41 ± 0.01	0.51 ± 0.01
MPs	55.20 ± 0.36	1.08 ± 0.07	1.75 ± 0.05	5.60 ± 0.05	0.17 ± 0.00

## 2 Experimental design, materials and methods

### 2.1 Preparation of MPs using UPPS

MPs were prepared by a self-developed apparatus, UPPS (Fig. 1) [[2], [3], [4], [5]]. The principle of UPPS to prepare MPs was realized by compressing the liquid into micro-droplets through high-speed rotating disk, and subsequent micro-droplets solidification with solvent evaporation under the airflow field. Finally, the dry powder can be collected from the collection plate.

### 2.2 Influence of formulation compositions on the drug release behavior of EC based MPs

#### 2.2.1 Drug release profiles of EC based MPs loading various additives

To investigate whether the solid-liquid interface phenomenon and the drug release rule were affected by the physicochemical properties of additives, the drug release profiles of EC based MPs loading HPMC/PEG/F127 as additives were compared. All the MPs were prepared by UPPS at a feeding rate of 7.8 ml/min and a rotating speed of 9000 rpm. The drug release tests were performed according to the paddle method III of ChP 2015 (50 rpm, 37.0 ± 0.5 °C) with a ZRS-8G dissolution tester (TDTF, Tianjin, China). 200 ml of deionized water, or simulated gastrointestinal fluid (pH shift release, the first two hours of release in 150 ml pH 1.2 HCl, followed by shift to pH 6.8 PBS with addition of 50 ml 0.2 M phosphate sodium) was used as the dissolution medium.

Interestingly, the drug release rate of all formulations (Fig. 2) was dramatically reduced in HCl comparing to PBS and deionized water, excluding the influence of interaction between EC and additives. Therefore, the media-dependent drug release profile of MPs could be as a function of different interaction between media and MPs, especially the wettability of MPs by media, which was further investigated.

#### 2.2.2 Drug release profiles of EC MPs loading different model drugs

To investigate the influence of drug on the release performance of EC MPs, ketoprofen or ciprofloxacin hydrochloride loaded EC MPs were prepared by UPPS as the same protocol used for preparing HupA loaded EC MPs. The dissolution test was conducted as described in Section 2.2.1, and was carried out in 200 ml of pH 1.2 HCl, pH 6.8 PBS, deionized water, or simulated gastrointestinal fluid.

The influence of physicochemical properties of drug on the release profiles of EC MPs were displayed in Fig. 3. Contrary to HupA, ketoprofen is a weak acid drug, which has a significantly higher solubility in pH 6.8 PBS than in pH 1.2 HCl. Therefore, ketoprofen conferred inferior wettability to MPs compared with HupA, which resulted in a slower drug release and a more serious drug release prohibition effect in HCl (Fig. 3A). Likewise, the drug release rate in different medium followed the same trend to HupA loaded EC MPs. However, conditions were somewhat different for the water-soluble drug, like ciprofloxacin hydrochloride (Fig. 3B). The drug release prohibition effect in HCl was absent and the corresponding drug release rate was even faster than deionized water, revealing that the drug release and wettability of EC MPs might be influenced by the acid component present in the drug.

### 2.3 The powder characteristics of the optimized MPs

The powder characteristics (flow energy, permeability and compressibility) of Ludiflash® and MPs were determined by FT4 Powder Rheometer (FreemanTechnology, Tewkesbury, UK). The main parameters obtained from the flow energy test were displayed in Table 1, and denoted as the following.

#### 2.3.1 Flow energy

The flow energy denotes the workforce required to move the blade through the powder from the top to the bottom of the vessel. A stabilized energy is achieved through seven identical “compete” test cycles.

(1) The Stability Index (*SI*) is the factor used to assess whether the flow energy changes during repeating tests and is calculated as follows.

$$\mathrm{SI}=\frac{\text{Flow}\;\text{energy}\;\text{of}\;\text{test}\;7}{\text{Flow}\;\text{energy}\;\text{of}\;\text{test}\;1}$$

The *SI* of Ludiflash® and MPs was 1.03 and 1.08, respectively. They were approximate to 1. It indicated that both Ludiflash® and MPs showed stable powder characteristic with good reproducibility of tests.

(2) The Basic Flow Energy (*BFE*) corresponds to the stabilized energy of test 7, which is required to displace a conditioned powder sample during downwards testing. Since the *BFE* value was dominated by the gravity, Ludiflash® had remarkably higher *BFE* value than MPs, which may be ascribed to the greater conditioned bulk density (*CBD*) of Ludiflash® than MPs.
(3) The Flow Rate Index (*FRI*) is the factor representing the flow energy change with the blade tip speed at a decreasing factor of 10 [6]. It can evaluate the sensitivity of powders to the blade speed and can be calculated using the following equation.

$$\mathrm{FRI}=\frac{\text{Flow}\;\text{energy}\;\text{of}\;\text{test}\;11\left(10\text{mm/s}\right)}{\text{Flow}\;\text{energy}\;\text{of}\;\text{test}\;8\left(100\text{mm/s}\right)}$$

Generally, cohesive powders are more sensitive to changes in flow rate and have higher *FRI*. The *FRI* of Ludiflash® and MPs was 1.27 and 1.75, respectively, which demonstrated that MPs was less free-flowing than Ludiflash®. Additionally, the *FRI* of powders is above 1 because the flow energy and voids/contact between particles are virtually dependent on the flow rate [6].

(4) The Specific Energy (*SE*) represents the energy per gram required to displace the conditioned powder during upwards testing. It indicates the flowability of the powder in an unconfined or low stressed environment and provides information on the relative cohesion level of powders [6,7]. The higher *SE* value is an indication of lager cohesion because *SE* is strongly affected by the cohesive and mechanical interlocking forces between particles [6]. The SE of Ludiflash® and MPs was 5.41 and 5.60, respectively, suggesting that MPs had inferior flowability to Ludiflash®. The *SE* value between 5∼10 revealed that MPs had moderate cohesion [8].

#### 2.3.2 Permeability

Permeability is a measurement of how easily the air can be transmitted in the material. The pressure drop of MPs and Ludiflash® increased with the increase of the given stress. The permeability is reduced because particles become more tightly packed at higher normal stress. Compared to Ludiflash®, MPs had lower pressure drop at the same given normal stress. Therefore, it was more permeable. The good permeability of MPs is beneficial to direct compression since the air can be easily expelled out of the voids during compression.

#### 2.3.3 Compressibility

Compressibility is defined as the volume change under given normal stress. The volume change of Ludiflash® and MPs increased with the increasing stress since the powders were more conditioned at higher stress. The compressibility index (CI) is calculated as the ratio of compressed density measured at 15 KPa to the bulk conditioned density at no consolidation [9]. The CI of Ludiflash® and MPs was 11.3% and 20.4%, respectively. The higher compressibility of MPs illustrated that they were less free-flowing than Ludiflash®, which was consistent with the results of free energy and permeability tests.
